# Vergleich der Effektivität von multiplen dynamischen Behandlungsstrategien unter Nutzung der Target-Trial-Emulierung: Kontrafaktischer Ansatz zur Kausalinferenz aus Real-World-Daten

**DOI:** 10.1007/s11553-023-01033-8

**Published:** 2023-06-12

**Authors:** Felicitas Kuehne, Lára Hallsson, Marjan Arvandi, Sibylle Puntscher, Beate Jahn, Gaby Sroczynski, Uwe Siebert

**Affiliations:** 1 Institute of Public Health, Medical Decision Making and Health Technology Assessment, Department of Public Health, Health Services Research and Health Technology Assessment, UMIT TIROL – University for Health Sciences and Technology, Eduard-Wallnoefer-Zentrum 1, 6060 Hall in Tirol, Österreich; 2 Center for Health Decision Science and Departments of Epidemiology and Health Policy & Management, Harvard T.H. Chan School of Public Health, 677 Huntington Ave, 02115 Boston, MA USA; 3 Institute for Technology Assessment and Department of Radiology, Massachusetts General Hospital, Harvard Medical School, Boston, MA USA

**Keywords:** Kontrafaktischer Ansatz, Realdaten, Kausale Inferenz, Kausaldiagramme, g‑Methoden, Counterfactual approach, Real-world data, Causal inference, Causal diagrams, g-methods

## Abstract

**Hintergrund:**

Therapieentscheidungen, die durch „Wenn-dann“-Algorithmen basierend auf bspw. Krankheitsverläufen oder vergangenen Therapien geregelt werden, sind dynamische Fragestellungen. Die Effektivität von dynamischen Therapiestrategien wird häufig mit Real World Data (RWD), d. h. Realdaten, untersucht. Einerseits bieten RWD ein großes Potenzial, da hiermit viele unterschiedliche in der Routineversorgung vorkommende Therapiestrategien analysiert werden können. Andererseits bergen Effektschätzer aus RWD-Analysen ein hohes Verzerrungspotenzial.

**Ziel der Arbeit:**

Dieser Artikel beschreibt, wie dynamische Behandlungsstrategien mithilfe von RWD adäquat verglichen und damit die optimale Therapiestrategie identifiziert werden können.

**Material und Methoden:**

Wir beschreiben, wie die Kombination aus drei Ansätzen eine kausale Interpretation der Ergebnisse erlaubt. Hierzu gehören (1) Kausaldiagramme, (2) Target-Trial-Emulierung sowie (3) statistische g‑Methoden. Der beschriebene kausale Ansatz und die genannten Begriffe und Konzepte werden erläutert und anhand eines Fallbeispiels verdeutlicht, in welchem untersucht wird, wann die antivirale Therapie bei behandlungsnaiven Patient:innen mit HIV-Infektion begonnen werden sollte.

**Ergebnisse:**

Kausaldiagramme visualisieren kausale Prozesse, die der Datengenerierung zugrunde liegen. Sie helfen, Parameter zu identifizieren, die in der Analyse berücksichtigt werden müssen. Die Target-Trial-Emulierung simuliert eine randomisierte klinische Studie, indem alle möglichen dynamischen Strategien definiert, die Patientendaten kopiert („geklont“) und jede:r Patient:in jedem Behandlungsarm zugewiesen werden. In einer kausalen Per-Protokoll-Analyse werden alle Patient:innen, die das jeweilige Protokoll einer Behandlungsstrategie verletzen, zensiert. Durch g‑Methoden wird für informatives Zensieren adjustiert. Die erwarteten Outcomes jeder Behandlungsstrategie werden simuliert und miteinander verglichen.

**Schlussfolgerung:**

Dynamische Behandlungsstrategien können mithilfe von RWD adäquat verglichen werden, wenn drei kausale Ansätze kombiniert werden und die erforderlichen Daten vorliegen. Diese Ansätze sind (1) Kausaldiagramme, (2) Target-Trial-Emulierung sowie (3) statistische g‑Methoden.

## Abstract (en)

**Background:**

Treatment decisions that are dependent on if–then rules on disease status or prior treatment information are dynamic treatment decisions. The effectiveness of dynamic treatment strategies is often investigated with real-world data (RWD). As many different therapy strategies can be observed in routine practice, RWD offer great potential. However, RWD are always associated with risks for several biases including immortal time and selection bias.

**Objectives:**

This article shows how to adequately compare dynamic treatment strategies and identify the optimal strategy. A case study is used to illustrate the causal approach described above.

**Materials and methods:**

We describe how the combination of three counterfactual approaches allows causal interpretation of results. We describe causal diagrams, target trial emulation, and g-methods. The described causal approach is illustrated by a case study examining when antiviral therapy should be initiated in treatment-naïve patients with human immunodeficiency virus (HIV) infection.

**Results:**

Causal diagrams visualize underlying causal processes. They help to identify parameters that need to be considered in the analysis. Target trial emulation simulates a randomized clinical trial by defining all possible dynamic strategies, copying (“cloning”) patient data, and assigning each patient to each treatment arm. In a causal per protocol analysis, all patients violating the protocol of a given treatment strategy are censored. Informative censoring is adjusted by g-methods. The expected outcomes of each treatment strategy are simulated and compared.

**Conclusions:**

Dynamic treatment strategies can be adequately compared using RWD when three causal approaches are combined, and the necessary data are available. These approaches are (1) causal diagrams, (2) target trial emulation, and (3) statistical g-methods.

## Hinführung zum Thema

Real World Data (RWD), d. h. Realdaten, bilden die Realität im Behandlungsalltag inklusive unterschiedlicher, in der Praxis eingesetzter Behandlungsmöglichkeiten ab. In den Daten liegt daher großes Potenzial, um die Effektivität verschiedener dynamischer Behandlungsstrategien miteinander zu vergleichen. Hierzu zählen Fragestellungen, wann eine Therapie begonnen, beendet, unterbrochen oder modifiziert werden soll und in welcher Reihenfolge Therapien aufeinander folgen sollten. Die Nutzung solcher longitudinaler RWD zur Gewinnung kausaler Rückschlüsse (in der Epidemiologie als „Kausalinferenz“ bezeichnet) aus den Beobachtungsdaten, weist auch bekannte Probleme, wie Confounding, Immortal-Time-Bias und Selektionsfehler auf. Ansätze, wie Kausaldiagramme und Target-Trial-Emulierungen, gepaart mit g‑Methoden helfen, das Potenzial der RWD auszuschöpfen, bei gleichzeitiger Kontrolle systematischer Verzerrungen (in der Epidemiologie als „Bias“ bezeichnet). Diese Begriffe und Konzepte werden in diesem Artikel vorgestellt und diskutiert.

## Motivation

Um eine hochwertige medizinische Versorgung zu gewährleisten, sollten Entscheidungen zur Versorgung von Patient:innen auf der zum entsprechenden Zeitpunkt vorhandenen besten Evidenz basieren. Zur Zulassung auf dem deutschen Markt durchlaufen Arzneimittel und Therapieformen in der Regel Studien, die ihren Nutzen und ihre Wirksamkeit nachvollziehbar und transparent belegen. Diese Studien sind vorwiegend randomisierte kontrollierte klinische Studien („randomized controlled trials“, RCT), die durch ihre Struktur systematische Fehler, wie Selektionsfehler beim Studieneinschluss und Confounding, vermeiden, damit eine hohe interne Validität besitzen und somit eine qualitativ hochwertige Evidenz liefern. Im klinischen Alltag sind die anstehenden Entscheidungen aber meistens sehr komplex und Erkenntnisse aus RCT spiegeln meist nur einen Teil der gesamten Lösung wider. Neben direkten Vergleichen der Effektivität zweier Therapien spielen in der Versorgungsforschung folgende Dinge eine wichtige Rolle: (1) Patient:innen entsprechen oft nicht der Studienpopulation einer klinischen Studie (d. h. limitierte externe Validität), (2) Krankheitsschwere oder Komorbiditäten und deren Behandlungen beeinflussen die Therapieentscheidung (Confounding), (3) Schaden wie z. B. Nebenwirkungen, Krankenhaustage etc. müssen dem Nutzen gegenübergestellt werden (Abwägungen), und (4) die Zeit in medizinischer Behandlung und die Lebensqualität spielen für die Patient:innen eine wichtige Rolle (patientenrelevante Outcomes).

Des Weiteren haben Therapien häufig eine dynamische Komponente. Dynamische Therapiestrategien basieren auf weiteren Informationen während des Krankheits- bzw. Therapieverlaufs. Solche Informationen sind z. B. Progression der Erkrankung, Nebenwirkungen der verabreichten Medikamente oder Informationen über andere (Begleit)therapien. Dynamische Therapiestrategien haben eine „Wenn-dann“-Komponente. Eine Fragestellung, wann eine Therapie begonnen, gestoppt, unterbrochen oder geändert werden sollte, basiert beispielsweise häufig auf der Schwere der Erkrankung oder Ereignissen während des Behandlungsverlaufs und kann anhand von bestimmten Biomarkern, einer Progression der Erkrankung, Komplikationen oder durch bestimmte Testergebnisse als Algorithmus definiert werden. Betrachtet man beispielsweise Patient:innen mit einer HIV-Infektion, stellt sich die Frage, wann eine antivirale Therapie optimaler Weise begonnen werden sollte. Dieser Zeitpunkt kann anhand von Schwellenwerten des CD4-Markers definiert werden, der den Immunstatus anzeigt. Die verschiedenen explizit definierten dynamischen Therapiestrategien können dann in einer RWD-Analyse miteinander verglichen werden.

Für die Beantwortung dieser beispielhaften Frage gibt es verschiedene Ansätze:

1. Ein RCT kann die verschiedenen dynamischen Behandlungsstrategien vergleichen [1]. Bei absoluter Therapie-Compliance und ohne „loss to follow-up“ ist die Analyse von RCT unkompliziert, besitzt hohe interne Validität und bietet damit hochwertige Evidenz. Allerdings sind dynamische Behandlungsstrategien häufig auf einen längeren Zeitraum angelegt als die Dauer des Follow-up üblicher RCT und hätten u. U. sehr viele Arme. Aus diesen Gründen fehlen oft RCT für dynamische Therapien, da sie enorme Kosten verursachen oder nicht praktikabel bzw. unethisch sind.
2. Beobachtungsstudien mit einem ausreichend langen Follow-up bilden die tatsächliche Langzeitrealität im Behandlungsalltag ab. Das bedeutet, dass mit diesen Daten dynamische Behandlungen miteinander verglichen werden können [5, 8]. Die Analyse von Beobachtungsdaten birgt allerdings die Gefahr, dass systematische Fehler wie Confounding, Selektionsfehler oder Immortal-Time-Bias die Ergebnisse verzerren [35]. Die Evidenz von Beobachtungsstudien gilt daher in der Regel als schwach.
3. Dynamische Therapien können auch in entscheidungsanalytischen Modellierungen simuliert werden [45, 46]. Diese mathematischen Modelle bilden den Krankheitsverlauf und potenzielle Behandlungsalgorithmen ab, wie sie z. B. in der HIV-Therapie durchgeführt wurden [10, 26, 50]. Evidenz aus verschiedenen Quellen werden miteinander verknüpft und verschiedene (u. a. auch kombinierte) Endpunkte können für jede Behandlungsstrategie ermittelt und verglichen werden. Ein entscheidungsanalytisches Modell ist jedoch immer eine Vereinfachung der Realität mit Annahmen und kann nur so gut sein wie die Evidenz, die in dieses Modell eingeht.

In diesem Artikel zeigen wir, wie ein kausaler Ansatz unter Berücksichtigung des Target-Trial-Prinzips in der Analyse von Beobachtungsdaten helfen kann, dynamische Behandlungsstrategien valide auf ihre Effekte hin zu vergleichen und damit die optimale Interventionsstrategie zu identifizieren.

## Herausforderungen bei dynamischen Behandlungen

Behandlungsfragen in der Praxis unterscheiden sich häufig von den Fragestellungen eines RCT, auf deren Grundlage die Zulassung erfolgte. Patient:innen haben evtl. Nebenerkrankungen. Neben der Effektivität spielen andere Faktoren eine wichtige Rolle, wie z. B. die Zeit mit der Familie, behandlungsfreie Zeit, Medikamentennebenwirkungen, weitere Behandlungsoptionen bei Therapieversagen etc. Daraus ergeben sich typische dynamische Fragestellungen, wie beispielsweise:

1. Wann ist der optimale Zeitpunkt, um mit der Therapie zu beginnen?
2. Wann soll eine Therapie unterbrochen oder beendet werden?
3. Was ist das optimale Intervall zwischen Medikamenteneinnahmen?
4. Welche Therapiereihenfolge ist optimal? Sollte zwischen zwei Therapien eine Pause eingehalten werden?
5. Wann sollte welche Dosisveränderung vorgenommen werden?

Für die Analyse von dynamischen Therapieeffekten bieten Beobachtungsdaten viele Möglichkeiten. Behandlungsstrategien, die auf den Erfahrungen der Mediziner:innen beruhen und angewandt werden, können beobachtet und analysiert werden. Andererseits ist die Analyse von RWD nicht einfach [4]. Die Entscheidung für den einen oder anderen Behandlungsansatz ist nicht zufällig. Sie werden durch Faktoren beeinflusst, die evtl. auch gleichzeitig das Outcome beeinflussen (Confounding), wie z. B. den Schweregrad der Erkrankung. Wenn die Behandlung von einem Faktor beeinflusst wird, der gleichzeitig das Outcome beeinflusst und die Therapie wiederum diesen Faktor beeinflusst, was bei der Symptomatik einer Erkrankung der Fall sein kann, spricht man von zeitabhängigem Confounding. Hierbei ist der Confounder gleichzeitig ein Intermediärschritt zwischen Therapie und Outcome. Ferner ist bei Beobachtungsstudien eine Zuordnung zu den Vergleichsarmen häufig komplex, da die Daten meist nur die tatsächliche Behandlung enthalten, nicht aber die Behandlungsintension. Stirbt eine Person, ohne behandelt worden zu sein, weiß die analysierende Person nicht, ob diese Person am nächsten Tag behandelt werden sollte und mit welcher Therapie. Aus diesem Grund ist ein sog. kausaler kontrafaktischer Ansatz notwendig.

## Kontrafaktischer Ansatz

Der kontrafaktische Ansatz basiert auf der Idee, dass eine beobachtete Handlung ein beobachtetes Outcome und weitere hypothetische, kontrafaktische Outcomes hat. Ein kontrafaktisches Outcome ist das hypothetische Outcome der nicht beobachteten Handlung. Wird beispielsweise beobachtet, dass eine Person täglich raucht und nach 10 Jahren stirbt, ist ein kontrafaktisches Outcome das hypothetische Outcome, das dieser Person widerfahren wäre, wenn dieselbe Person nicht geraucht hätte, aber alle anderen Parameter beim Start der Beobachtung gleichgeblieben wären. Bei verschiedenen (Be‑)Handlungen gibt es mehrere kontrafaktische Outcomes. In dem genannten Beispiel ist ein weiteres kontrafaktisches Outcome das Resultat, das man beobachtet hätte, wenn dieselbe Person nach 2 Jahren aufgehört hätte zu rauchen.

Beim kontrafaktischen Ansatz wird Kausalität nachgewiesen, indem ein beobachteter Zustand nach einer Handlung mit dem hypothetischen Zustand verglichen wird, bei dem eine andere Handlung stattgefunden hätte. Es wird also z. B. eine „Welt“ mit einer Therapie mit einer identischen „Welt“, aber ohne die Therapie verglichen. In RCT werden durch die Randomisierung die beiden identischen Welten simuliert bzw. angenähert. In einer „Welt“ (einem Vergleichsarm) findet die Intervention statt, in der anderen „Welt“ (dem anderen Vergleichsarm) nicht [34]. Auch in Analysen von Beobachtungsdaten können wir identische Welten simulieren. Hierbei müssen folgende Bedingungen erfüllt sein:

1. Die Populationen der Behandlungsstrategien müssen vergleichbar sein und keine systematischen Unterschiede aufweisen. Bei der Prüfung dieser Annahme helfen Kausaldiagramme [14, 49].
2. Die Struktur einer prospektiven Analyse muss gewährleistet werden. Hierbei unterstützt das Konzept der Target-Trial-Emulierung [7, 18, 19, 24, 32].
3. Systematische Verzerrungen durch Confounding müssen weitgehend vermieden werden. Bei Baseline-Confounding können traditionelle statistische Methoden wie Regression, Propensity Scores etc. herangezogen werden [30]. Bei zeitabhängigem Confounding müssen Kausalmethoden (g-Methoden) wie z. B. „marginal structural models“ (MSM), g‑Formula oder g‑Estimation angewandt werden [38–40, 42, 44].

In den folgenden Abschnitten nutzen wir als Fallbeispiel die Fragestellung, wann die antivirale Therapie bei behandlungsnaiven Patient:innen mit HIV-Infektion begonnen werden sollte, um den kontrafaktischen Ansatz bei Beobachtungsdaten zu erläutern. Im Zuge dessen beschreiben wir Kausaldiagramme, die sog. gerichteten azyklischen Graphen („directed acyclic graph“, DAG), die Target-Trial-Emulierung sowie die analytischen Methoden „inverse probability weighting“ (IPW) mit MSM, die parametrische g‑Formula sowie g‑Estimation mit „structural nested models“ (SNM).

### Gerichteter azyklischer Graph

**Abb. 1:**
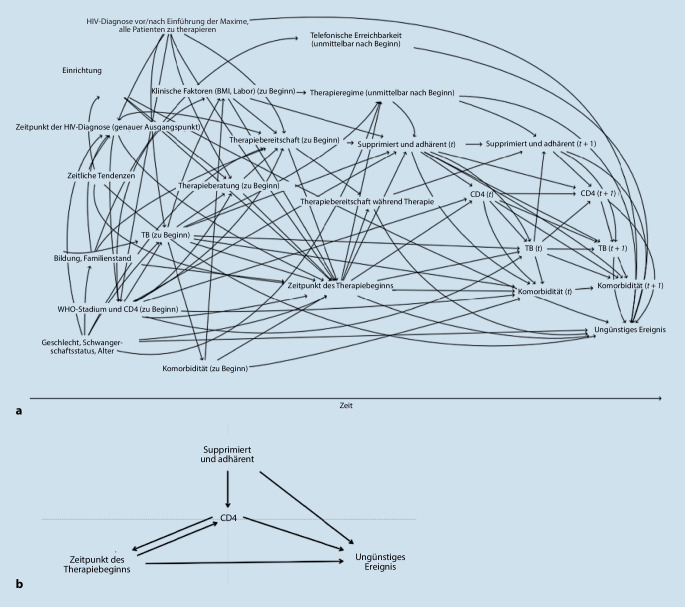
**a** Gerichteter azyklischer Graph (DAG, „directed acyclic graph“) zum Therapiebeginn von antiviraler HIV-Therapie von Kerschberger et al. ([22]; *BMI* Body Mass Index, *t* Time, *TB* Tuberkulose, *HIV* „human immunodeficiency virus“). **b** Vereinfachung des DAG als Kausalgraph. (**a** von Kerschberger et al. [22], publiziert unter der CC By 4.0 Lizenz https://creativecommons.org/licenses/by/4.0/, Abbildung ins Deutsche übersetzt; **b** Vereinfachung nach Kerschberger et al. [22])

Die DAG fassen Daten zusammen und visualisieren natürliche kausale Prozesse unabhängig davon, welche Daten erhoben wurden. Alle Variablen, die Teil des kausalen Prozesses sind, werden abgebildet. Pfeile zwischen den Variablen bzw. fehlende Pfeile stellen Kenntnisse oder Annahmen von kausal gerichteten Zusammenhängen dar. Ein Pfeil von A nach Y symbolisiert die Kenntnis oder Annahme, dass die Ausprägung der Variablen A die Ausprägung der Variablen Y verursacht. Die eigentliche Annahme liegt im Fehlen eines Pfeils, was bedeutet, dass kein kausaler Zusammenhang zwischen den jeweils zwei Variablen angenommen wird. Dies kann eine sehr starke Annahme sein. Ein vorhandener Pfeil symbolisiert einen Effekt, wobei dieser Effekt sehr klein bzw. fast nicht vorhanden sein kann. Kerschberger et al. haben einen DAG erstellt, um die Wirkung der sofortigen antivirale HIV-Therapie auf ein ungünstiges Behandlungsergebnis (Verlust der Nachbeobachtung, Tod, Virusversagen, Behandlungswechsel) abzuschätzen ([22]; Abb. 1).

Die DAG helfen festzustellen, ob eine kausale Frage beantwortet werden kann. Dies wird als Identifizierbarkeit (des kausalen Effekts anhand der gemessenen Variablen) bezeichnet. Hierbei sind drei verschiedene Variablentypen bzw. -konfigurationen zu unterscheiden, die in DAG sichtbar sind: (1) Ein Confounder ist eine Variable, die sowohl die Intervention oder Handlung als auch das Outcome direkt oder indirekt beeinflusst, (2) ein Collider ist eine Variable, die durch zwei andere Variablen verursacht wird und (3) ein Mediator ist eine Variable, die auf dem kausalen Pfad zwischen zwei Variablen liegt. Abb. 1b zeigt, dass die Variable CD4

1. ein Confounder zwischen „Zeitpunkt des Therapiebeginns“ (ZTB) und „ungünstiges Ereignis“ (UE) ist; ZTB ← CD4 →UE,
2. ein Collider zwischen ZTB und „supprimiert und adhärent“ (SA); ZTB → CD4 ← SA und
3. ein Mediator zwischen ZTB und UE; ZTB → CD4 →UE ist.

Der Graph unterstützt die Identifikation des Biomarkers CD4 als zeitabhängigen Confounder. Bei zeitabhängigem Confounding sind g‑Methoden in der statistischen Analyse notwendig [14, 16, 41, 48].

### Target-Trial-Emulierung

Der Ansatz der Target-Trial-Emulierung ist ein struktureller Ansatz [4]. Durch das Klonen der Daten wird der kontrafaktische Ansatz simuliert, d. h. es werden parallele Welten nachgebildet. Hierbei werden die Erfahrungen mit RCT genutzt, welche mit den Beobachtungsdaten möglichst nah „nachgeahmt“ (emuliert) werden. Die analysierende Person erstellt für den „target trial“ (d. h. einem hypothetischen RCT zur gleichen Fragestellung) ein Studienprotokoll, das sog. Target-Trial-Protokoll. Dieses hypothetische RCT wird aber aus ethischen, praktischen, ökonomischen, zeitlichen oder anderen Gründen nicht durchgeführt. Im Anschluss daran wird das Protokoll an die Beobachtungsdaten angepasst. Das Studienprotokoll legt dazu Einschlusskriterien, Behandlungsstrategien, deren Zuordnung, Outcomes, Studienbeginn, Follow-up und den statistischen Analyseplan fest und wird dann auf die Daten angewandt [18, 23]. Da in den meisten Routinedaten eine Intention-to-treat-Variable fehlt, wird häufig eine kausale Per-Protokoll-Analyse durchgeführt [17]. Hierbei werden bei den Individuen bzw. deren Kopien die Daten zum Zeitpunkt der Verletzung des Target-Trial-Protokolls zensiert, d. h. Ereignisse und andere Daten nach diesem Zeitpunkt werden in der Analyse nicht mehr berücksichtigt. Dies kann zu einem Bias führen, wenn das zur Zensierung führende Ereignis nicht zufällig auftritt, sondern systematische Gründe hat, welche zu einer Verzerrung des Ergebnisses führen könnten. (Dies wird informative Zensierung genannt.) Ein Bias durch informative Zensierung wird durch adäquate statistische Methoden wie „inverse probability of censoring weighting“ (IPCW) vermieden. Informell ausgedrückt, werden hierbei verlorengegangenen Daten der zensierten Personen ersetzt durch Daten ähnlicher nicht-zensierter Personen (die ein größeres Gewicht bekommen) und damit der Datenverlust möglichst unverzerrt korrigiert.

Für die Evaluation von dynamischen Therapieeffekten eignet sich die Analyse von RWD anhand des Target-Trial-Ansatzes besonders bzw. ist hierfür evtl. sogar erforderlich [5, 24, 51]. RWD zeigen Variabilität in den Behandlungsstrategien, da verschiedene Ärztinnen und Ärzte unterschiedliche Entscheidungen treffen, auch wenn sie ähnliche Patientengeschichten sehen. Diese Variabilität und die daraus resultierende Information in Beobachtungsdaten kann mit Hilfe des „target trials“ zur Erkenntnisgewinnung genutzt werden. Im Target-Trial-Protokoll können die Vergleichsarme frei gewählt werden, solange sie in der Realität vorkamen und die entsprechenden Daten verfügbar sind. Ethische, pragmatische oder ökonomische Aspekte schränken die Anzahl der Vergleichsarme nicht ein.

**Abb. 2:**
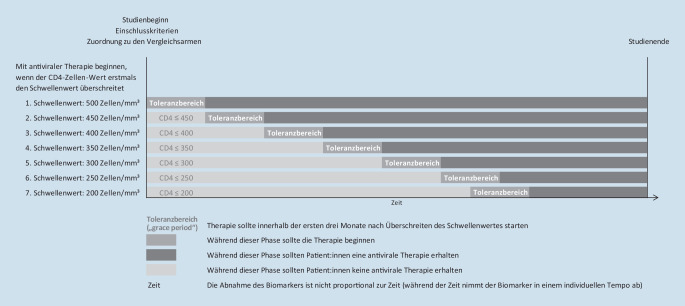
Vergleichende Behandlungsstrategien im „target trial“

Die Behandlungsstrategien sind durch einfache Regeln definiert, die auf Informationen u. a. zum Krankheitsverlauf oder zu einer anderen Behandlung basieren. Im Fallbeispiel wird die Frage nach dem optimalen Zeitpunkt der antiviralen HIV-Therapie in Bezug auf CD4-Zellenanzahl untersucht, d. h. der Fokus des Interesses von Cain et al. ist der Therapiebeginn [5, 6]. Die möglichen dynamischen Behandlungsstrategien werden definiert als „Antiviraler Therapiebeginn, sobald die CD4-Zellzahl das erste Mal unter × Zellen/mm^3^ fällt“, wobei die Variable × verschiedene Werte zwischen 200 und 500 in Schritten von 10 bzw. 50 annimmt. In einem RCT würden nur Patient:innen eingeschlossen werden, die jeder dieser Behandlungsstrategien zugeordnet werden könnten. Das heißt in diesem Fall, dass Patient:innen eingeschlossen werden können, die einen CD4-Wert von mindestens 500 Zellen/mm^3^ haben. Somit werden auch in den Target-trial-Personen eingeschlossen, die eine CD4-Zellzahl von mindestens 500 Zellen/mm^3^ zeigen. Die Daten dieser Personen werden kopiert. Anschließend werden die hypothetischen Patient:innen jedem Vergleichsarm zugewiesen. In den unterschiedlichen Behandlungsarmen werden Patient:innen dann ab Einschluss in die Studie beobachtet. Sie bringen ihre Personenzeit in jedem Vergleichsarm ein. Abb. 2 zeigt eine schematische Darstellung der Behandlungsarme.

Ein Individuum kann Information oder Personenzeit in mehrere Behandlungsarme gleichzeitig einbringen. Tab. 1 zeigt, dass alle Patient:innen zum Zeitpunkt t = 0 konform mit den Protokollen aller Strategien sind. Mit der Zeit ändern sich sowohl der Behandlungszustand als auch der CD4-Wert. Damit sind manche der Kopien der Patient:innen nicht mehr unbedingt konform mit den Protokollen bestimmter Behandlungsstrategien. Patient:in 1 startet z. B. die Therapie im ersten Monat bei einem CD4-Wert von 489 Zellen/mm^3^. Damit verhält er/sie sich nur mit Strategie 1 konform. Patient:in 5 fängt in den ersten 4 Monaten gar keine Therapie an. Da er/sie im 4. Monat das erste Mal einen CD4-Wert < 300 Zellen/mm^3^ hat, verletzt er/sie in diesem Monat das erste Mal das Protokoll von Strategie 5–7.

**Tab. 1:** Hypothetische Patient:innen, die den verschiedenen Behandlungsprotokollen folgen

ID	Zeit in Monaten	CD4-Wert	Behandlung 1 = ja; 0 = nein	Im Einklang mit Protokoll der Strategie mit CD4-Schwellenwert (Zellen/mm^3^) 1 = ja; 0 = nein (ohne Grace-Periode)							Im Einklang mit Protokoll der Strategie mit CD4-Schwellenwert (Zellen/mm^3^) 1 = ja; 0 = nein (mit Grace-Periode [1 Monat])						
				500	450	400	350	300	250	200	500	450	400	350	300	250	200
1	0	527	0	1	1	1	1	1	1	1	1	1	1	1	1	1	1
1	1	489	1	1	0	0	0	0	0	0	1	0	0	0	0	0	0
1	2	430	1	1	0	0	0	0	0	0	1	0	0	0	0	0	0
1	3	356	1	1	0	0	0	0	0	0	1	0	0	0	0	0	0
1	4	201	1	1	0	0	0	0	0	0	1	0	0	0	0	0	0
2	0	502	0	1	1	1	1	1	1	1	1	1	1	1	1	1	1
2	1	464	0	0	1	1	1	1	1	1	1	1	1	1	1	1	1
2	2	405	0	0	0	1	1	1	1	1	0	1	1	1	1	1	1
2	3	331	1	0	0	1	1	0	0	0	0	1	1	1	0	0	0
2	4	176	1	0	0	1	1	0	0	0	0	1	1	1	0	0	0
3	0	518	0	1	1	1	1	1	1	1	1	1	1	1	1	1	1
3	1	480	0	0	1	1	1	1	1	1	1	1	1	1	1	1	1
3	2	421	1	0	1	0	0	0	0	0	1	1	0	0	0	0	0
3	3	347	1	0	1	0	0	0	0	0	1	1	0	0	0	0	0
3	4	192	1	0	1	0	0	0	0	0	1	1	0	0	0	0	0
4	0	514	0	1	1	1	1	1	1	1	1	1	1	1	1	1	1
4	1	476	0	0	1	1	1	1	1	1	1	1	1	1	1	1	1
4	2	417	0	0	0	1	1	1	1	1	0	1	1	1	1	1	1
4	3	343	0	0	0	0	0	1	1	1	0	0	1	1	1	1	1
4	4	188	1	0	0	0	0	1	1	1	0	0	1	1	1	1	1
5	0	510	0	1	1	1	1	1	1	1	1	1	1	1	1	1	1
5	1	472	0	0	1	1	1	1	1	1	1	1	1	1	1	1	1
5	2	413	0	0	0	1	1	1	1	1	0	1	1	1	1	1	1
5	3	339	0	0	0	0	0	1	1	1	0	0	1	1	1	1	1
5	4	184	0	0	0	0	0	0	0	0	0	0	0	0	1	1	1

Beobachtungsdaten weisen eine Variabilität des Therapiestarts nach dem oben beschriebenen Schwellenwert auf. Patient:innen beginnen die Therapie evtl. nicht unmittelbar nach Verordnung, sondern z. B. wegen persönlicher Terminplanungen erst einen Monat später. Diese Patient:innen sollten im Target Trial dennoch als protokollkonform eingestuft werden. Hilfreich ist daher die Verwendung einer sog. „Grace-Periode“ oder „Toleranzbereich“, d. h. eine Frist, innerhalb derer die Therapie beginnen sollte und noch als protokollkonform bezeichnet werden kann. Cain et al. definieren bspw. eine Grace-Periode von 3 Monaten [11, 23, 29]. Das Protokoll definiert, dass die Therapie innerhalb von 3 Monaten nach erstmaligem Unterschreiten des jeweiligen CD4-Zellzahlschwellenwertes beginnen soll. Wenn in Tab. 1 eine sog. Grace-Periode von 1 Monat erlaubt wird, werden beispielsweise bei Patient:in 4 die Protokolle der Behandlungsstrategien 5–7 auch im 4. Monat nicht verletzt.

Die für den „target trial“ wichtigen Daten müssen durch die verfügbaren RWD abgebildet werden. Hierbei ist die inhaltliche, d. h. diagnostische Einteilung, ebenso wie die zeitliche Zuordnung für die Analyse von Bedeutung. Die Analyse des „target trial“ für dynamische Behandlungsstrategien benötigt zeitabhängige Daten zu Therapie, Confounder und Outcome. Die Zeit wird für die Analyse in hypothetische Intervalle unterteilt. Für die Analyse ist die Information der Parameter innerhalb dieser Intervalle wichtig. Wenn zu den hypothetischen Zeitpunkten keine neuen Datenpunkte vorliegen, müssen Annahmen getroffen werden. Im Beispiel vom Beginn der HIV-Therapie kann die Annahme gemacht werden, dass dem behandelnden Arzt nur die Information über den CD4-Zellenwert oder Komorbiditäten etc. bis zu dem jeweiligen Zeitpunkt vorliegen. Hier würde für die Analyse der Realität entsprechend die Information der historischen Daten fortgeschrieben werden, bis neue Informationen hinzukommen. Wenn im Gegensatz zu der exemplarisch behandelten Fragestellung das kontinuierliche Monitoring eine Rolle spielt, kann die Annahme gemacht werden, dass fehlende neue Information auf keinen Doktor-Patient:innen-Kontakt hinweist.

**Abb. 3:**
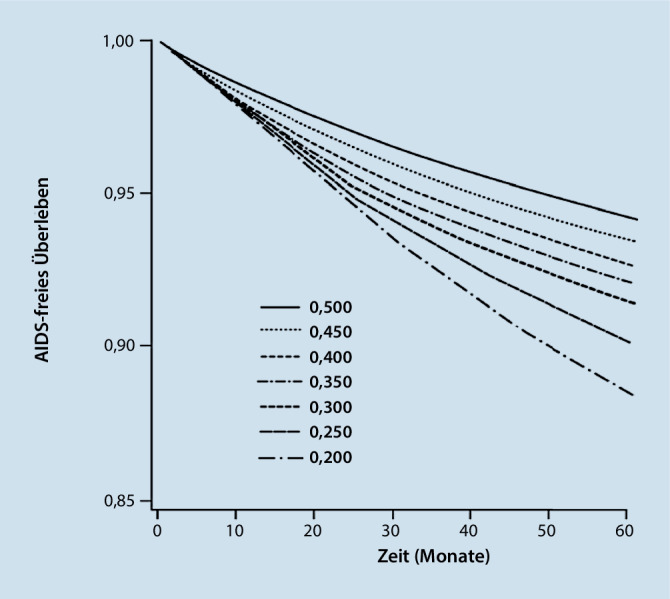
AIDS-freies („acquired immune deficiency syndrome“) Überleben bei einer kombinierten antiretroviralen Therapie beginnend bei CD4-Zellzahlschwellenwerten von 0,200–0,500 × 109 Zellen/l (Used with permission of [American College of Physicians—Journals], from [6]; permission conveyed through Copyright Clearance Center, Inc.)

Als Ergebnis eines „target trial“, der dynamische Therapien vergleicht, können die hypothetischen Outcomes eines jeden Therapiearms berechnet und gegenübergestellt werden. Im Beispielfall des optimalen Zeitpunktes der antiviralen HIV-Therapie wurden von den Autoren der Studie die hypothetischen Überlebenskurven simuliert und dargestellt (Abb. 3; [6]).

### g-Methoden

Beim Vorliegen von zeitabhängigem Confounding sind ggf. g‑Methoden einzusetzen [14, 16, 41, 48]. Der Begriff „g-Methoden“ weist auf die Tatsache hin, dass diese Methoden „generalisierte“ Methoden darstellen im Sinne von Anwendbarkeit auf beide Confounding-Typen – zeitunabhängiges Confounding und zeitabhängiges Confounding. In den folgenden Abschnitten werden drei g‑Methoden kurz beschrieben.

### IPCW

Das IPCW wird angewandt, um für informative Zensierung der Daten zu kontrollieren, d. h. einen Bias zu eliminieren, der durch einen Ausschluss (Zensierung) bestimmter Personenzeiten zu nicht (mehr) vergleichbaren Gruppen führte. Zu jedem Zeitpunkt der Zensierung wird der Einfluss der unzensierten Daten anhand von Gewichten erhöht. So wird eine Pseudopopulation kreiert und die Analyse simuliert die Assoziation, die beobachtet worden wäre, wären alle Individuen in der Studie geblieben.

Im ersten Schritt wird das sogenannte statistische Gewichtsmodell berechnet [31, 37, 47]. Hierzu wird für jedes Intervall die Wahrscheinlichkeit einer Zensierung geschätzt. Im Fall der Analyse von Cain et al. wird die kovariatenabhängige Wahrscheinlichkeit berechnet, dass mit der Therapie begonnen wird. Aus diesen Wahrscheinlichkeiten werden für jedes Intervall Gewichte berechnet. Das Gewicht ist die inverse kovariatenabhängige Wahrscheinlichkeit, bis zu dem gegebenen Zeitpunkt nicht zensiert worden zu sein. Im zweiten Schritt wird im gewichteten Outcome-Modell für jede Strategie die Wahrscheinlichkeit des Outcomes geschätzt und über alle Strategien verglichen [31, 37, 47]. Um die optimale Behandlungsstrategie zu finden, wird eine kontinuierliche Korrelation zwischen den Strategien und dem Outcome modelliert.

### g-Formula

Auch mit Hilfe der parametrischen g‑Formula wird eine kontrafaktische Welt simuliert [38, 40, 44]. Hierbei wird der Gesamteffekt einer Behandlungsstrategie in mehreren Schritten sequenziell zusammengesetzt. Zunächst werden die Daten genutzt, um über die Zeit für das Outcome sowie für jede Kovariate ein parametrisches Modell nach Konditionierung für vergangene Variablenwerte eines jeden Individuums anzupassen. Im zweiten Schritt wird mit Hilfe dieser Modelle eine Monte-Carlo-Simulation durchgeführt und dabei für jede der verglichenen Behandlungsstrategien die Therapievariable entsprechend der jeweiligen Behandlungsstrategie „gesetzt“. Dann kann das Ergebnis jeder Strategie aus diesem hypothetischen Datensatz abgelesen werden.

In unserem Fallbeispiel der Feststellung des optimalen HIV-Therapiebeginns wird im Behandlungsstrategiearm mit dem Schwellenwert 350 CD4-Zellen/mm^3^ der Therapieparameter in jedem Zeitintervall, in dem der CD4-Wert 350 Zellen/mm^3^ oder mehr annimmt, künstlich auf den Wert 0 (keine Therapie) gesetzt. Alle Kovariaten sowie das Outcome werden mit Hilfe der im Schritt 1 definierten parametrischen Modelle simuliert. So werden alle kontrafaktischen Therapiearme simuliert, und die entsprechenden kontrafaktischen Outcomes können miteinander verglichen werden.

### g-Estimation

Die g‑Estimation simuliert die kontrafaktische Welt mit Hilfe eines kausalen Modells für den Einfluss der Therapie auf das Outcome auf Personenebene (SNM). Die Parameter dieses Modells können dann als kausale Effekte interpretiert werden, wenn – informell ausgedrückt – die daraus resultierende kontrafaktische Welt unabhängig von der realen Welt ist [39].

Im Beispiel des optimalen Beginns der HIV-Therapie würde ein Therapieeffekt mithilfe eines kausalen Modells simuliert. Mit diesem Modell werden für jede Patient:in und für verschiedene Modellparameter die kontrafaktischen Outcomes berechnet. Anschließend werden die Modellparameter unter Berücksichtigung der Confounder so lange variiert, bis das Kausalmodell kontrafaktische Outcomes generiert, die keine Rückschlüsse darauf zulassen, welche der HIV-Therapiestrategien der jeweilige Patient bzw. die jeweilige Patientin tatsächlich bekommen hat. Dies entspricht mathematisch der Annahme von keinem ungemessenen Confounding („no unmeasured confounding“, NUC).

### Limitationen und Annahmen

Die g‑Methoden eignen sich gut, um mehrere (dynamische) Behandlungsstrategien kausal zu vergleichen und für zeitabhängiges Confounding zu adjustieren. Die drei vorgestellten Methoden basieren auf Modellen und die Validität der Ergebnisse beruhen damit auf korrekten Modellspezifikationen. Alle drei vorgestellten Methoden fußen auf der Annahme von NUC. Die drei Methoden basieren zudem auf Modellen. Dies bedeutet, dass die Validität der Ergebnisse damit auch von der Korrektheit der Modellspezifikationen abhängt.

Die IPCW benötigt zwei Modelle, ein Gewichtsmodell und ein Outcome-Modell. Die g‑Formula beinhaltet ein Modell für jede zeitabhängige Kovariate und für das Outcome. Die Korrektheit der Ergebnisse einer g‑Formula hängen besonders stark von der korrekten Modellspezifität ab. Die g‑Estimation benötigt ein valides kausales Outcome-Modell in Abhängigkeit von der Therapie. Die Struktur des Modells muss richtig gewählt sein, um eine aussagekräftige Schätzung des Therapieeffekts zu erhalten. Die Korrektheit der Ergebnisse eines Modells mit IPCW basieren u. a. stark auf der Annahme der Positivität. Positivität verlangt über alle Kovariaten-Strata hinweg eine positive Wahrscheinlichkeit aller potenziellen Behandlungsstrategien.

## Anforderungen an die Daten

Um dynamische Behandlungsstrategien mit Beobachtungsdaten analysieren und vergleichen zu können, müssen die Beobachtungsdaten u. U. sowohl inhaltlich sehr umfangreich als auch über einen ausreichen langen Zeitraum vorhanden sein. Zum einen müssen alle Parameter, die im DAG als relevant für die Identifizierbarkeit des Kausaleffekts deklariert wurden, gemessen und im Datensatz vorhanden sein. Wenn diese Parameter über die Zeit variieren, müssen sie auch über die Zeit gemessen und dokumentiert sein. Idealerweise sind die Zeitabstände zwischen den Datenpunkten möglichst klein. Je größer der Abstand zwischen den Datenpunkten, desto größer ist das Risiko für verbleibendes („residuelles“) zeitabhängiges Confounding.

Faktoren, die zu Zensierungen im IPCW führen können, müssen bei allen Patient:innen gemessen sein. Es reicht nicht aus, diese Information nur von den Patient:innen vorliegen zu haben, die zensiert werden, sondern sie muss auch von den Patient:innen vorliegen, die nicht zensiert werden. In unserem Fallbeispiel gibt es prognostische Faktoren, die den Beginn der HIV-Therapie beeinflussen, also zeitabhängige Confounder. Diese Faktoren müssen daher im Gewichtsmodell berücksichtigt werden und für alle Personen gemessen sein.

Wie in allen Beobachtungsstudien müssen die Daten valide gemessen sein. Häufig werden Sekundärdaten von Krankenkassen zur Gewinnung neuer Evidenz genutzt [21]. Hierbei spiegeln die Daten z. B. die Abrechnung wider und nicht immer den exakten zeitlichen und diagnostischen Ablauf der Ereignisse. Weitere Diskussionen mit Expert:innen, Transparenz über die Annahmen sowie Sensitivitätsanalysen sind notwendig, um valide kausale Rückschlüsse ziehen zu können und die Akzeptanz von kausalen Analysen zu fördern.

## Ausblick

Der Target-Trial-Ansatz bietet eine Möglichkeit, dynamische Behandlungsstrategien, die in Beobachtungsdaten dokumentiert sind, zu analysieren und die Ergebnisse valide kausal zu interpretieren. Wie weit sich dieser Ansatz etabliert, hängt von mehreren Faktoren ab.

1. Wie in diesem Artikel dargestellt, sind die Anforderungen an die genutzten Daten groß. Einerseits wächst im Zeitalter der Digitalisierung der Umfang an Daten an, andererseits handelt es sich um sensible Daten, die besonders geschützt werden müssen. Die Debatten um Gesundheitsdaten und deren Verfügbarkeit finden vermehrt statt.
2. Die Möglichkeit, Daten aus verschiedenen Quellen zu verknüpfen ist Gegenstand aktueller Initiativen. Die Datenverknüpfung bietet großes Potenzial, eine solide und umfangreiche Basis für Beobachtungsstudien mit RWD zu bieten [36].
3. Der Target-Trial-Ansatz wurde 2016 das erste Mal explizit unter diesem Begriff veröffentlicht [12, 18, 19]. In den letzten Jahren wurde dieser Ansatz vermehrt angewandt. Durch die klare Struktur und verständliche Sprache wächst die Kenntnis über diesen Ansatz.
4. Zur Stärkung der Akzeptanz des Target-Trial-Ansatzes werden häufig RCT mit Beobachtungsdaten emuliert. Studien zeigen, dass hierbei neben dem Studiendesign auch die Merkmale der Studienkohorte simuliert werden müssen [25, 27, 29, 43].
5. Target-Trial-Protokolle sollten vorab veröffentlicht werden, um die Akzeptanz dieses Ansatzes und die Transparenz zu stärken [23].
6. Die Anwendbarkeit des Target-Trial-Ansatzes bei großen Datenbanken ist eine Herausforderung. Künstliche Intelligenz wird in kausale Methoden integriert und hilft, Parameter aus großen Datenbanken zu ermitteln [3]. Da künstliche Intelligenz häufig auf Prädiktionen basiert, müssen deren Anwendung in kausalen Analysen genau durchdacht sein und die hier vorgestellten Kausalprinzipien berücksichtigt werden [15].
7. Als erste Health Technology Assessment (HTA)-Organisation hat im Juni 2022 das National Institute for Clinical Excellence (NICE) das Konzept des Target-Trial-Ansatzes in seinem „real-world evidence framework“ aufgenommen. Es ist zu erwarten, dass weitere HTA-Organisationen diesem Beispiel folgen werden [32].

## Weitere Beispiele aus Gesundheitsförderung und/oder Prävention

Im Bereich der Primärprävention gibt es Beispiele aus der jüngsten Vergangenheit mit erfolgreicher Anwendung des TTE-Ansatzes mit Klonen – Zensierung – Gewichtung und Einsatz der g‑Methoden bei Beobachtungsstudien mit RWD wie z. B. der Bestimmung der Real-world-Effektivität bzw. der Optimierung der COVID-19-Impfung („coronavirus disease 2019“; [2, 28]). Im Bereich der Sekundärprävention werden diese Methoden bereits seit einigen Jahren eingesetzt und führten zu wichtigem Erkenntnisgewinn wie z. B. beim Dickdarmkrebsscreening [13]. Auch bei der Verwendung von RWE zur medikamentösen Prävention von negativen Folgen des Hormonmangels [20] oder nach Infektion mit Hepatitis C [4, 33] wurden die Methoden erfolgreich eingesetzt. Im Bereich der Gesundheitsförderung wurden der Target-trial-Ansatz erst jüngst eingesetzt wie z. B. im Jahr 2021 in der Beobachtungsstudie der American Heart Association zu „dietary goals and mortality“ [9]. Zusammenfassend ist anzumerken, dass der Target-Trial-Ansatz in Kombination mit modernen validen kausalen Auswertungsmethoden erst eine relativ kurze Geschichte hat und derzeit noch nicht breit für RWE-Studien im Bereich Prävention und Gesundheitsförderung eingesetzt wird. Angesichts der Entwicklungen in den letzten Jahren ist davon auszugehen, dass sich dies in den kommenden Jahren ändern wird und der Einsatz der genannten Methoden von den Entscheidungsträgern zunehmend gefordert wird als Voraussetzung für valide RWE. Damit gehört der Einsatz und Erfahrungsgewinn der genannten Konzepte zu den wichtigsten zukünftigen Feldern in der Versorgungsforschung zu Prävention und Gesundheitsförderung.

## Fazit für die Praxis

- Real World Data (RWD) aus Beobachtungsstudien bieten großes Potenzial, die Effektivität dynamischer Behandlungsstrategien zu untersuchen: RWD zeigen viele verschiedene Behandlungsmöglichkeiten auf, beinhalten repräsentative Daten und sind häufig sowohl im großen Umfang als auch über einen langen Zeitraum verfügbar.
- Der Target-Trial-Ansatz gepaart mit Kausaldiagrammen und g‑Methoden hilft das Potenzial der RWD auszuschöpfen bei gleichzeitiger Kontrolle der systematischen Fehler, wie Confounding, Immortal-Time-Bias und Selektionsfehler.
- Der Target-Trial-Ansatz ist ein kontrafaktischer Ansatz, RWD zu analysieren: Die zu vergleichenden Strategien werden klar definiert; die Daten werden so häufig kopiert, wie es verschiedene Behandlungsstrategien gibt. Jede Person wird jedem Behandlungsarm zugewiesen. Anschließend wird eine kausale Per-Protokoll Analyse wird durchgeführt, indem jede Person zu dem Zeitpunkt zensiert wird, zu dem er/sie das Protokoll verletzt.
- g‑Methoden kontrollieren für informative Zensierung und stellen die kausale Interpretierbarkeit der Ergebnisse sicher.
